# The Therapeutic Potential of EGCG and Pro-EGCG in Mitigating Ovarian Hyperstimulation Syndrome: Unraveling the Modulatory Mechanism through the VEGF Pathway

**DOI:** 10.7150/ijbs.98653

**Published:** 2025-04-22

**Authors:** Sijia Wang, Lanlan Fang, Luping Cong, Qiongqiong Jia, Waner Wu, Mingpeng Zhao, Tinchiu Li, Jacqueline Pui Wah Chung, Ka Kei Fung, Vivian Ching Man Lam, Yingpu Sun, Jung-Chien Cheng, David Yiu Leung Chan

**Affiliations:** 1 Assisted Reproductive Technology Unit, Department of Obstetrics and Gynecology, Faculty of Medicine, The Chinese University of Hong Kong, Hong Kong SAR, 999077, China.; 2 Center for Reproductive Medicine, Henan Key Laboratory of Reproduction and Genetics, The First Affiliated Hospital of Zhengzhou University, Zhengzhou, 450003, China.; 3 The Vancouver Prostate Centre, Department of Urologic Sciences, University of British Columbia, Vancouver, BC, Canada.

**Keywords:** OHSS, EGCG, VEGF pathway, OHSS animal model, RNA-Seq

## Abstract

Ovarian hyperstimulation syndrome (OHSS) is a severe complication of controlled ovarian hyperstimulation (COH) during *in vitro* fertilization (IVF) treatment, characterized by increased capillary permeability. Vascular endothelial growth factor (VEGF) is a key mediator in OHSS, with serum VEGF levels correlating with its severity. In this study, we investigated the therapeutic potential of (-)-epigallocatechin-3-gallate (EGCG) and its derivative, Pro-EGCG, in mitigating OHSS. Using both *in vitro* and *in vivo* models, including primary human granulosa-lutein cells, the human granulosa-like tumor KGN cell line, and a rat OHSS model induced with pregnant mare serum gonadotropin, we found that EGCG and Pro-EGCG significantly reduced OHSS progression. This was supported by histological analyses, reductions in ovarian weight, and decreased VEGF expression at both transcriptomic and proteomic levels. Mechanistic studies revealed that EGCG and Pro-EGCG inhibit TGF-β-induced VEGF production through suppression of the TGF-β/Smad and PKA-CREB signaling pathways. RNA sequencing further validated the downregulation of VEGF expression following treatment. These findings highlight the potential of EGCG as a novel adjuvant therapy for managing OHSS, providing a mechanistic basis for its clinical application.

## Introduction

Ovarian Hyperstimulation Syndrome (OHSS) is a severe complication associated with assisted reproductive technologies (ARTs), particularly in *in vitro* fertilization (IVF) procedures^1^. It is characterized by excessive ovarian enlargement, often accompanied by the formation of multiple ovarian cysts and increased vascular permeability. This leads to fluid extravasation into the abdominal and pleural cavities 2, resulting in significant morbidity and, in severe cases, life-threatening complications. The severity of OHSS varies widely, with symptoms ranging from mild to severe. The reported incidence rate ranges from 0.5% to 5%, depending on individual conditions and the treatment protocols employed^3^. Common clinical symptoms include abdominal pain, bloating, and respiratory distress. Severe cases may present with ascites, pleural effusions, and electrolyte imbalances^4^. Although the precise etiology of OHSS remains unclear, it is primarily attributed to the stimulation of multiple ovarian follicles by exogenous gonadotropins. This overstimulation triggers excessive vascular endothelial growth factor (VEGF) release, which increases capillary permeability^5^.

VEGF plays a critical role in angiogenesis and vascular permeability^6^,^7^. Elevated VEGF levels, mainly originating from hyperstimulated ovaries, increase vascular permeability, causing fluid leakage into the peritoneal and pleural cavities. This leads to characteristic OHSS symptoms such as abdominal distension, ascites, and respiratory distress^8^. VEGF and its receptors are expressed in granulosa cells of preovulatory follicles and granulosa-lutein cells of the corpus luteum 9-11. Notably, VEGF levels in follicular fluid surpass those in serum and correlate with OHSS severity^12^,^13^. Previous studies have shown that human chorionic gonadotropin (hCG) increases VEGF expression in human granulosa cells and elevates serum VEGF concentrations^14^-^17^. Significantly, both animal models and human studies have highlighted the therapeutic potential of targeting VEGF or its receptors to prevent OHSS development^18^,^19^.

Epigallocatechin gallate (EGCG), a major bioactive polyphenol found in green tea, has garnered significant attention due to its diverse biological effects and potential therapeutic applications in female reproductive diseases, including polycystic ovary syndrome (PCOS), endometriosis, and uterine fibroids^20^,^21^. EGCG is known for its antioxidant, anti-inflammatory, and anti-cancer properties, as well as its ability to modulate cell signaling pathways involved in cell proliferation, apoptosis, and angiogenesis 22. Studies have demonstrated that EGCG inhibits VEGF and its receptor expression in pathological processes such as gastric, ovarian, and breast cancers 23-26. EGCG octaacetate (Pro-EGCG ), a modified form of EGCG, enhances its stability and bioavailability *in vivo* 27. However, there is limited research on the effects of EGCG on women experiencing OHSS during IVF treatment, and the specific mechanisms through which EGCG exerts its effects on OHSS remain unclear. In this study, we investigated the effects of EGCG on OHSS and its underlying molecular mechanisms using *in vitro* and *in vivo* models.

Our findings show that EGCG or its prodrug, Pro-EGCG, attenuates OHSS development in a rat model. In KGN cells and primary human granulosa-lutein (hGL) cells, both EGCG and Pro-EGCG significantly reduce VEGF and VEGFR-2 expression. Furthermore, EGCG inhibits TGF-β-induced VEGF production by suppressing the canonical SMAD signaling pathway. Additionally, EGCG downregulates VEGF expression via the 67-kDa laminin receptor-mediated PKA-CREB pathway. These findings provide new insights into the regulatory mechanisms underlying pathological angiogenesis in OHSS and suggest that EGCG and its derivatives could serve as potential therapeutic agents for managing this condition.

## Materials and methods

### Cell cultures and reagents

The KGN cell line, a human granulosa-like tumor cell line, possesses functional follicle-stimulating hormone receptors, and it was obtained from the laboratory of Prof. Weiyi Chen by courtesy at the Chinese University of Hong Kong. Primary human granulosa-lutein (hGL) cells were isolated via density centrifugation from follicular fluid collected from women undergoing oocyte retrieval at the IVF laboratory of the Prince of Wales Hospital, Hong Kong. Both KGN and hGL cells were cultured in DMEM/F12 medium (Gibco) medium supplemented with 10% charcoal/dextran-treated fetal bovine serum (FBS) (Gibco), along with 100 U/mL penicillin and 100 μg/mL streptomycin (Gibco). The cell cultures were maintained at 37 °C in a humidified atmosphere containing 5% CO2. Recombinant human TGF-β1 was procured from R&D Systems. The pregnant mare serum gonadotropin (PMSG) was obtained from Solarbio. Antibodies against caspase-3 and the 67-kDa laminin receptor were purchased from Abcam, VEGF antibody was acquired from Thermo Fisher Scientific, Antibodies against phospho-CREB, CREB, SMAD2, phospho-SMAD2, SMAD3, and phospho-SMAD3 were obtained from Cell Signaling Technology. Detailed information regarding the antibodies used for western blot analysis in this study is provided in Supplemental Table 1.

### Human follicular fluid collection

The studies involving clinical samples received approval from the Joint Chinese University of Hong Kong - New Territories East Cluster Clinical Research Ethics Committee (CREC Ref No: 2018.533) for the collection of human follicular fluid and the primary granulosa cells. Infertile women meeting specific criteria were enrolled in the study after providing written informed consent between September 2021 and December 2022. Detailed information about the ovarian stimulation protocols is provided in a previous study^28^. The inclusion criteria were as follows: women aged 20-35 years with a BMI of 19-24.9, regular menstrual cycles, tubal factor infertility or male factor infertility, and no complications such as diabetes or abnormal thyroid function. Exclusion criteria included polycystic ovarian syndrome (PCOS), endometriosis, diminished ovarian reserve, chromosomal abnormalities, or hydrosalpinx. Follicular fluid was collected during oocyte retrieval, and hGL cells were isolated and purified using density centrifugation.

### Cell intervention

KGN and hGL cells were cultured in 6-well plates (1 × 10⁶ cells/well) or 12-well plates (5 × 10⁵ cells/well). When the cells reached approximately 80% confluence, they were treated with various concentrations of EGCG (Sigma-Aldrich, #E4143) or Pro-EGCG (Abcam, #ab145182) or dimethyl sulfoxide (DMSO) (Sigma-Aldrich). DMSO was used as the vehicle control, as both EGCG and Pro-EGCG were dissolved in DMSO. To maintain consistency, the control group received the same volume of DMSO as the experimental groups. After treatment, cells were harvested for protein and RNA extraction. To investigate the effects of EGCG on the function of TGF-β, KGN cells were pre-treated with EGCG (10 µM, 25 µM) for 24 h, followed by treatment with recombinant human TGF-β.

### Cell viability and proliferation assays

KGN and hGL cells were seeded into 12-well plates at a density of 5 × 10⁴ cells per well or 96-well plates at a density of 1 × 10⁴ cells per well. For cytotoxicity analysis, cells were allowed to reach approximately 80% confluence before being treated with varying concentrations of EGCG or Pro-EGCG in serum-free F12/DMEM medium for specified durations. For proliferation assays, treatments with varying concentrations of EGCG or Pro-EGCG were initiated immediately after seeding in F12/DMEM medium supplemented with 10% FBS and continued for the designated time points. For treatments exceeding 24 h, the culture medium was replenished daily with fresh drugs at the same concentrations.

Cell viability and proliferation were assessed using the MTT assay and cell counting methods. For the MTT assay, a solution of MTT in sterile PBS (5 mg/mL) was prepared and stored at 4 °C, protected from light. A 10 µL aliquot of this MTT solution was added to each well of a 96-well plate, followed by incubation at 37 °C in the dark for 4 hours. After incubation, 100 µL of DMSO was added to each well to dissolve the formazan crystals. Absorbance at 570 nm was measured using a spectrophotometric microplate reader. For cell counting, images were captured using a Leica microscope for analysis and documentation. Live and dead cells were counted in six random fields of view per well. The results were used to evaluate cell proliferation and viability under the specified treatment conditions.

### Western blotting analysis

Cells were harvested and lysed using RIPA lysis buffer (Cell Signaling Technology) containing 1% protease inhibitors (Boster) on ice. After centrifugation, the supernatant was carefully collected, and protein concentration was determined using a BCA assay kit (Bio-Rad). Equal amounts of protein were mixed with 5X SDS protein loading buffer (Solarbio) and heated at 100°C for 10 min. Proteins were separated by SDS-PAGE (Bio-Rad Laboratories) and transferred onto PVDF membranes (Bio-Rad Laboratories). Membranes were blocked for 1 h in 5% non-fat dry milk dissolved in Tris-buffered saline (TBS). After blocking, the membranes were incubated overnight at 4°C with primary antibodies diluted in 5% non-fat milk-TBS. Following primary antibody incubation, membranes were washed five times with TBS containing 0.1% Tween-20 (TBST). The membranes were then incubated with HRP-conjugated secondary antibodies for 1 h at room temperature. Immunoreactive bands were visualized using an enhanced chemiluminescent substrate (Bio-Rad Laboratories), and imaging was performed using a ChemiDoc MP Imager (Bio-Rad Laboratories).

### Reverse transcription-quantitative real-time PCR (RT-qPCR)

Total RNA was isolated and purified from cells collected at the designated time points using the RNeasy Mini Kit (Qiagen) or TRIzol reagent (Invitrogen), following the manufacturer's instructions. A 50 ng aliquot of RNA was reverse transcribed into first-strand complementary DNA (cDNA) using the cDNA Synthesis Kit (Takara Bio). Each 20 μL qPCR reaction contained 1X TB Green Premix Ex Taq (Takara Bio), 20 ng of cDNA, and 0.8 μM of the appropriate primer pairs. RT-qPCR was performed using an Applied Biosystems 7500 Fast Real-Time PCR System with a 96-well optical reaction plate. The primer sequences used are listed in Supplementary Table 1. Assay specificity was validated by performing a melting curve analysis and electrophoresis of the PCR products on an agarose gel. All RT-qPCR experiments were carried out in triplicate. Negative controls included water and RNA samples without reverse transcription. Relative mRNA quantification was calculated using the comparative Ct method, with GAPDH as the reference gene, applying the 2^-∆∆Ct^ formula.

### Small interfering RNA (siRNA) transfection

To downregulate endogenous mRNA expression, cells were transfected with 50 nM On-TARGETplus SMARTpool siRNAs targeting specific genes (Dharmacon). Transfection was performed using Lipofectamine RNAiMAX (Invitrogen), following the manufacturer's instructions. A siCONTROL Non-TARGETING pool siRNA (Dharmacon) was used as a control. After transfection, cells were incubated for 48 hours before proceeding with the experimental procedures. The efficacy of gene knockdown was assessed by western blot analysis.

### Rat OHSS model

Female Sprague-Dawley (SD) rats, aged three weeks and weighing between 32-36 g, were obtained from the Laboratory Animal Services Center at The Chinese University of Hong Kong for the study. The rats were housed in a controlled environment with free access to food and water. Ethical approval for the animal experiments was granted by The Chinese University of Hong Kong Animal Experimentation Ethics Committee.

A total of 32 female SD rats were randomly assigned to four experimental groups (n = 8 per group) using a computer-generated randomization sequence to minimize selection bias. The groups included a control group and three OHSS model groups. OHSS was induced based on a modified version of a previously established protocol to enhance reproducibility^29^. Specifically, 23-day-old SD rats received intraperitoneal injections of pregnant mare serum gonadotropin (PMSG) at 10 IU/day for four consecutive days. On the fifth day, human chorionic gonadotropin (hCG) was administered intraperitoneally at 10 IU to trigger ovulation. The control group received a single injection of PMSG (7 IU) on the third day, followed by hCG (10 IU) 48 hours later.

To assess the therapeutic effects of EGCG and Pro-EGCG, rats in the OHSS model groups were treated with either vehicle control (DMSO) or EGCG/Pro-EGCG (10 mg/kg, i.p. ) from day 4 to day 6. On the seventh day, all animals were euthanized under anesthesia, and peripheral blood, ascitic fluid, and ovarian tissue samples were collected for further analysis. Investigators responsible for sample collection and data analysis were blinded to the group allocations to minimize potential bias. Body weights were recorded every two days throughout the experiment to monitor the health status of the animals.

### Histological staining and immunohistochemistry (IHC) analysis

Ovarian tissue samples were obtained from distinct cohorts of both control and OHSS rats. The ovarian tissues were fixed in 4% paraformaldehyde solution (Sigma) and then embedded in paraffin. The embedded tissues were sectioned into 4-μm-thick slices. These sections underwent a series of preparatory steps, including deparaffinization in xylene, rehydration through an ethanol gradient, antigen retrieval in citrate buffer (pH 9.0) by microwave treatment, and blocking of endogenous peroxidase activity with 3% hydrogen peroxide. Next, the tissue sections were incubated overnight at 4°C with anti-rat VEGF antibody at a 1:100 dilution. Prior to antibody incubation, a blocking step was performed at room temperature for 20 min using 5% normal goat serum. After washing with PBS, the tissue sections were visualized using the DAB Substrate Kit (Dako, Denmark) and counterstained with hematoxylin. Images were acquired using a microscope and specialized software. The staining intensity was evaluated semi-quantitatively using the H-score method, which takes into account both the intensity of staining and the proportion of positively stained cells. The H-score was calculated using the formula: H-score = Σpi(i+1), where 'i' represents the staining intensity (1 = weak; 2 = moderate; 3 = strong), and 'pi' denotes the percentage of cells exhibiting each intensity (ranging from 0% to 100%). To ensure objectivity, two independent researchers, blinded to the experimental groups, performed the staining intensity assessment.

### Assessment of vascular permeability using Evans Blue dye

Vascular permeability was assessed in rats via intravenous injection of Evans Blue dye (Sigma-Aldrich, #E2129) following a standardized protocol. A 5% Evans Blue dye solution was prepared by dissolving 20 mg of dye in 1 mL of sterile saline and filtering it through a 0.22-μm syringe filter to remove particulates. Rats were restrained, and the tail was sterilized with 75% ethanol before injection. Using a 30 G needle attached to a 1 mL syringe, 100 μL of the prepared dye solution was administered into the tail vein. The dye was allowed to circulate for 30 min to ensure uniform vascular distribution. After the incubation period, the rats were euthanized by intraperitoneal injection of a ketamine-xylazine mixture, following guidelines approved by the Animal Experimentation Ethics Committee. To collect peritoneal fluid, 3 mL of sterile saline was injected into the abdominal cavity and allowed to rest for five min. A 1 mL aliquot of the peritoneal fluid was then collected, transferred to a 1.5 mL microcentrifuge tube, and mixed with 200 μL of 0.1 M NaOH to precipitate proteins. The samples were centrifuged at 2000 rpm for 10 minutes at 4°C, and the supernatant was transferred to a fresh tube. The absorbance of the supernatant was measured at 620 nm using a microplate reader. A standard curve was generated from known concentrations of Evans Blue dye to quantify the dye content in each sample. Vascular permeability was calculated and compared between experimental groups to evaluate differences in vascular leakage associated with OHSS.

### Measurement of rat serum VEGF: Enzyme-linked immunosorbent assay

The concentration of VEGF in rat serum samples was determined using an enzyme-linked immunosorbent assay (ELISA), following the manufacturer's instructions. The Rat VEGF ELISA kit was purchased from R&D Systems (#RRV00).

### Statistical analysis

Data are presented as the mean ± SEM from at least three independent experiments. Statistical analyses were performed using Prism 8 software. For comparisons between two groups, a t-test was used. For comparisons among multiple groups, a one-way analysis of variance (ANOVA) was performed, followed by Tukey's multiple comparison test. The assumptions were checked by assessing normality using the Shapiro-Wilk test. Effect sizes were measured using Cohen's d for t-tests and R² for ANOVA. A p-value of less than 0.05 was considered statistically significant.

## Results

### EGCG inhibits VEGF expression

**Figure 1:**
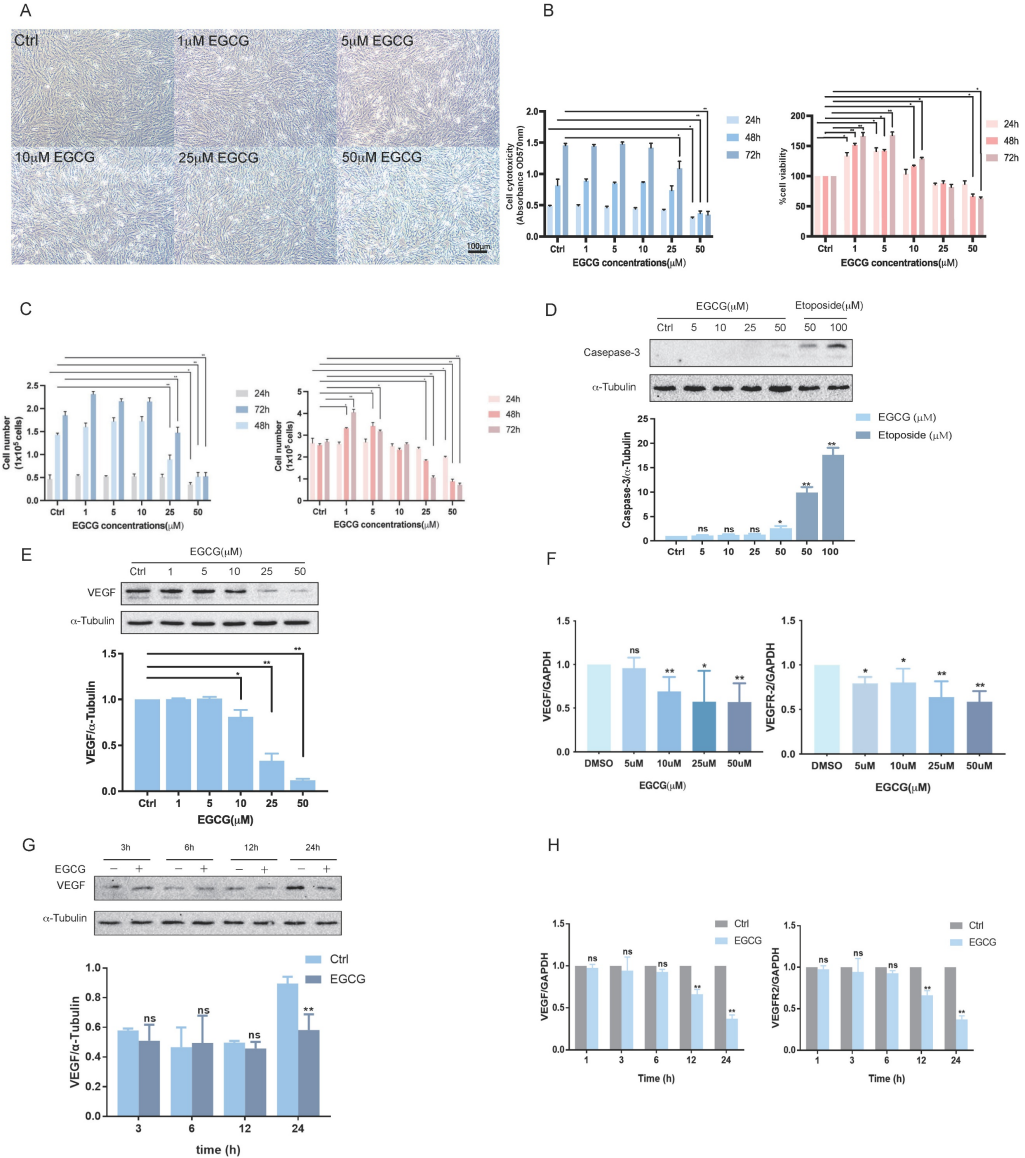
** EGCG inhibits VEGF expression. (A)** KGN cells were treated with vehicle control (DMSO) or various concentrations of EGCG (1 µM, 5 µM, 10 µM, 25 µM, and 50 µM) for 24 h, and cellular morphology was observed microscopically.** (B, C)** Cell viability (B) and proliferation (C) were evaluated via MTT assay and cell counting, respectively, following treatment with DMSO or EGCG at the indicated concentrations for 24, 48, and 72 h. **(D)** Western blot analysis of caspase-3 protein levels in KGN cells treated with DMSO or EGCG (5 µM, 10 µM, 25 µM, and 50 µM) for 24 h. Etoposide (50 µM and 100 µM) was included as a positive control. **(E, F)** VEGF protein expression (E) and VEGF/VEGFR-2 mRNA levels (F) were determined by western blot and RT-qPCR, respectively, in KGN cells treated with DMSO or EGCG at the specified concentrations for 24 h. **(G, H)** Time-course experiments were conducted to assess VEGF protein expression (G) and VEGF/VEGFR-2 mRNA levels (H). KGN cells were treated with DMSO or 25 µM EGCG for 3, 6, 12, and 24 h (G) or for 1, 3, 6, 12, and 24 h (H). Results are presented as the mean ± SEM from at least three independent experiments. Significant differences are denoted by asterisks (*p < 0.05, **p < 0.01).

It has been documented that, in physiological conditions, the serum concentration of EGCG remains below 1µM^30,31^. However, with EGCG supplementation, serum levels can elevate to as high as 7 µM^32^. To evaluate the effect of EGCG on Vascular Endothelial Growth Factor (VEGF), KGN cells were treated with EGCG at concentrations ranging from 1 to 50 µM. As shown in Figure 1A, treatment with varying EGCG concentrations for 24 hours did not significantly alter cell morphology. Cytotoxicity assays demonstrated that the inhibitory effect of EGCG on KGN cell proliferation depends on both concentration and duration. Similarly, the cytotoxic effect of EGCG on KGN cells showed a dose- and time-dependent pattern (Figure 1B). Lower concentrations of EGCG appeared to confer resistance to cytotoxic effects, which may be attributed to antioxidant properties. Cell counting provided additional validation, showing results consistent with those obtained from the MTT assay (Figure 1C). These findings suggest that at lower concentrations and shorter exposure times, EGCG within physiological limits does not induce cytotoxic effects in KGN cells. Caspase-3, a critical executioner caspase, plays a key role in the final stages of apoptosis, mediating DNA fragmentation, protein degradation, and membrane blebbing^33^. To confirm the apoptotic effect of EGCG in KGN cells, western blot analysis was performed to assess caspase-3 levels, with etoposide included as a positive control. As shown in Figure 1D, only treatment with 50 µM EGCG significantly increased caspase-3 expression, indicating apoptosis induction. Western blot analysis revealed that EGCG at concentrations of 1 and 5 µM had no effect on VEGF protein levels. In contrast, treatment with 10 µM, 25 µM, and 50 µM significantly downregulated VEGF protein expression after 24 hours (Figure 1E). These findings were supported by RT-qPCR results, which showed consistent inhibition of VEGF mRNA levels in KGN cells (Figure 1F). Previous research has highlighted the critical role of VEGF and its receptor VEGFR-2 in OHSS-related angiogenesis^19^. Interestingly, EGCG reduced VEGFR-2 mRNA levels at concentrations of 5 µM and higher following 24 hours of treatment (Figure 1F). To investigate the mechanisms underlying the effect of EGCG on VEGF expression, experiments were conducted using 25 µM EGCG. Western blot analysis confirmed significant inhibition of VEGF protein expression after 24 hours (Figure 1G). Additionally, RT-qPCR results showed that EGCG reduced VEGF and VEGFR-2 mRNA levels at both 12- and 24-hour time points (Figure 1H).

### 67LR-mediated PKA-CREB activation is required for the EGCG-reduced VEGF expression

**Figure 2:**
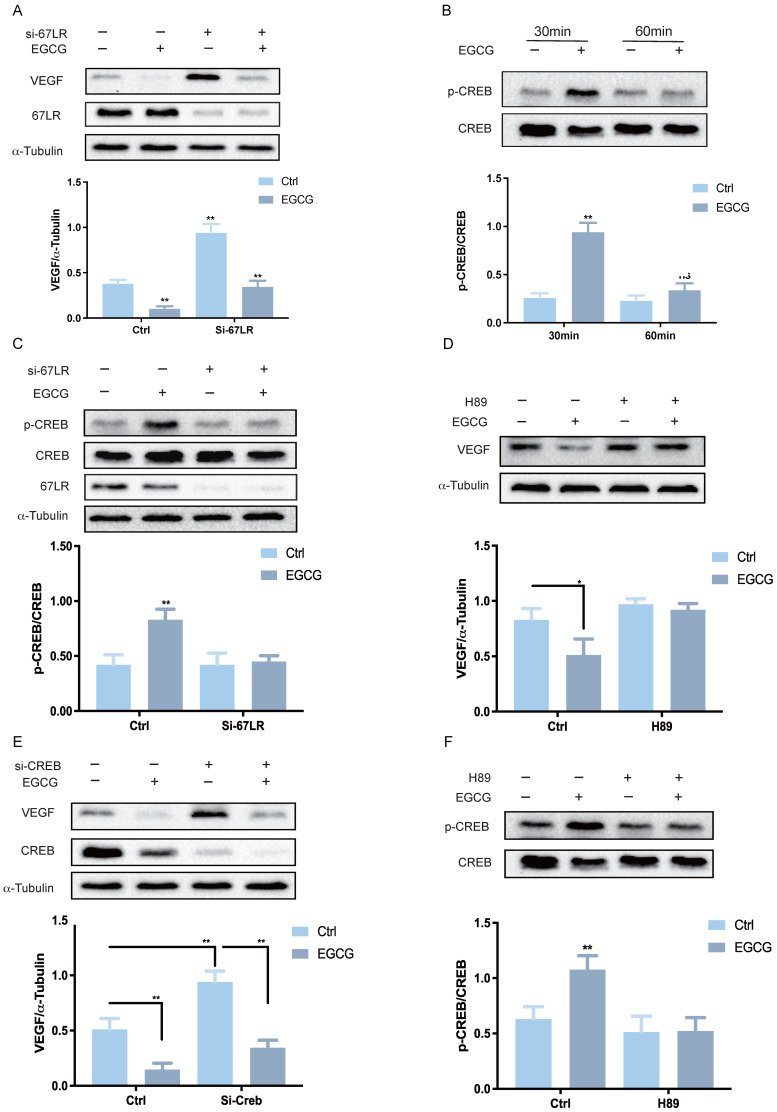
** 67LR-mediated PKA-CREB activation is required for EGCG-induced reduction in VEGF expression. (A)** KGN cells were transfected with 50 nM control siRNA (si-Ctrl) or 67LR siRNA (si-67LR) for 48 h, followed by treatment with 25 µM EGCG for 24 h. Protein levels of VEGF, 67-kDa laminin receptor (67LR), and α-Tubulin were analyzed by western blot. **(B)** Phosphorylated and total CREB protein levels were assessed via western blot in KGN cells treated with 25 µM EGCG for 30 or 60 min. **(C)** KGN cells were transfected with 50 nM si-Ctrl or si-67LR for 48 h and then exposed to 25 µM EGCG for 30 min. Protein levels of phosphorylated and total CREB, along with 67LR, were examined by western blot. **(D)** KGN cells were pre-treated with vehicle control (DMSO) or 1 µM H89 for 1 h before treatment with 25 µM EGCG for 24 h. Protein levels of VEGF and α-Tubulin were measured via western blot. **(E)** KGN cells were transfected with 50 nM si-Ctrl or CREB siRNA (si-CREB) for 48 h, followed by treatment with 25 µM EGCG for 24 h. Western blot analysis was performed to evaluate protein levels of VEGF, CREB, and α-Tubulin. **(F)** Phosphorylated and total CREB protein levels were evaluated by western blot in KGN cells pre-treated with DMSO or 1 µM H89 for 1 h, followed by treatment with 25 µM EGCG for 30 min. Results are presented as the mean ± SEM from at least three independent experiments. Significant changes are indicated by asterisks (*p < 0.05, **p < 0.01).

To investigate the molecular mechanism underlying EGCG-induced downregulation of VEGF expression, it is crucial to determine whether EGCG exerts its regulatory effects through direct intracellular entry or by interacting with a membrane receptor. The 67-kDa laminin receptor (67LR) has been well-documented as a membrane receptor for EGCG, mediating its physiological functions^34^. Previous studies have shown that 67LR is expressed in KGN cells and plays a critical role in EGCG-induced activation of the PKA-CREB signaling pathway, which promotes StAR expression and progesterone production in both KGN and hGL cells^35^. To evaluate the involvement of 67LR in EGCG-mediated VEGF suppression, specific siRNA targeting 67LR (si-67LR) was used to knockdown the endogenous 67LR mRNA levels in KGN cells. Knockdown of 67LR partially mitigated the inhibitory effects of EGCG on VEGF protein levels (Figure 2A). The protein kinase A (PKA)/cAMP-responsive element-binding protein (CREB) signaling cascade is a well-established pathway mediating VEGF expression downstream of 67LR. To examine the influence of EGCG on CREB activation, phosphorylated and total CREB levels were assessed by western blot after EGCG treatment. EGCG treatment increased CREB phosphorylation, with maximal activation observed at 30 min (Figure 2B).

In cells treated with si-67LR, EGCG-induced CREB activation was attenuated (Figure 2C). To further investigate the role of the CREB pathway in EGCG-mediated VEGF suppression, the PKA inhibitor H89 was employed. Pre-treatment of KGN cells with H89 abolished the suppressive effects of EGCG on VEGF protein levels (Figure 2D). To confirm the necessity of CREB and rule out potential off-target effects of pharmacological inhibitors, CREB expression was downregulated using specific siRNA (si-CREB). Knockdown of CREB inhibited the suppressive effect of EGCG on VEGF protein levels (Figure 2E). Finally, to confirm the role of PKA in the CREB pathway, KGN cells were pre-treated with H89, which significantly blocked the phosphorylation of CREB, as shown by western blot analysis (Figure 2F). These results collectively highlight the requirement of 67LR-mediated PKA-CREB activation for EGCG-induced downregulation of VEGF expression in KGN cells.

### EGCG inhibits VEGF expression via TGF-β signaling pathways

**Figure 3:**
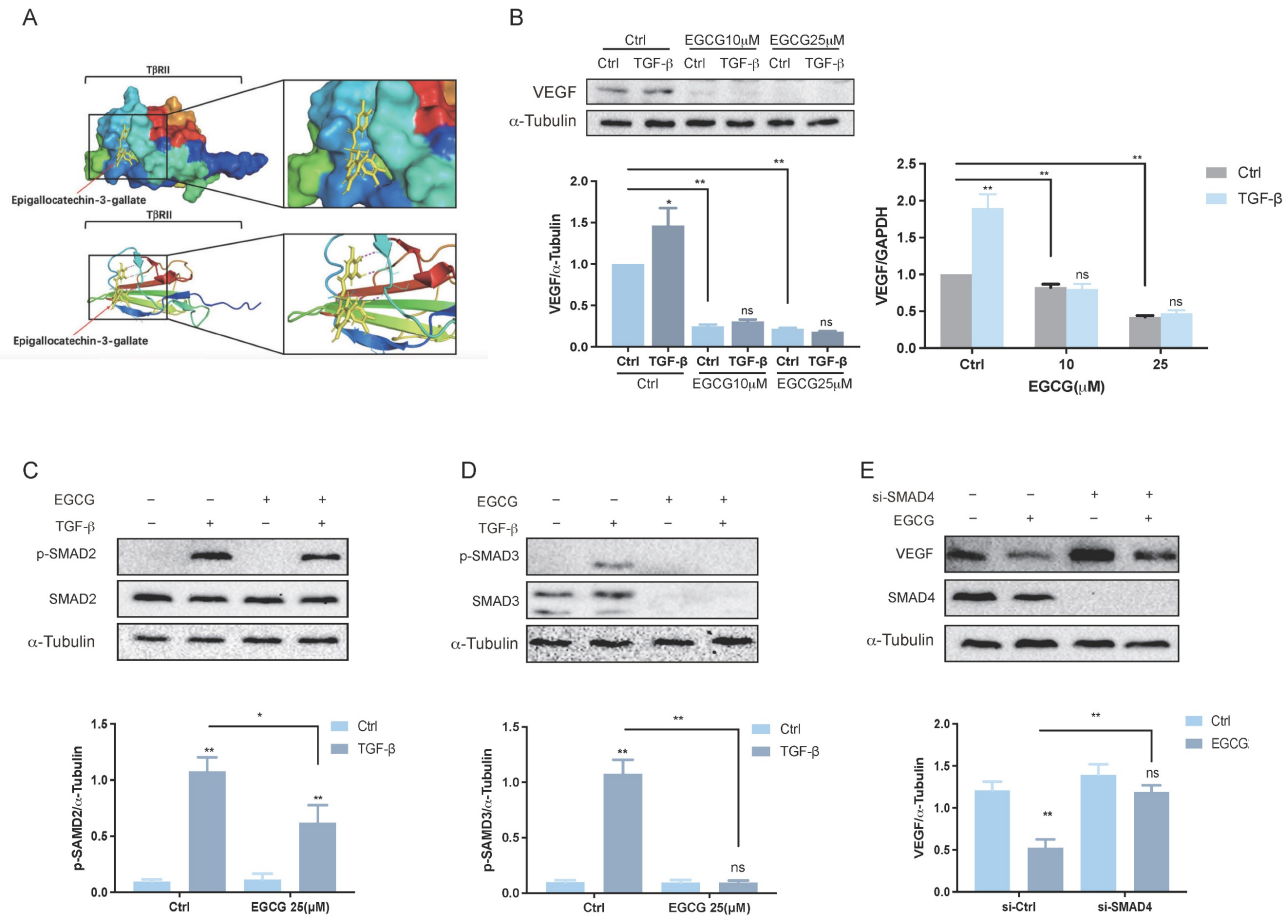
** EGCG inhibits VEGF expression via TGF-β signaling pathways. (A)** Molecular docking analysis demonstrates the interaction between EGCG and TGF-β receptor type II (TβRII). **(B)** KGN cells were pre-treated with vehicle control (DMSO), 10 µM EGCG, or 25 µM EGCG for 24 h, followed by stimulation with 5 ng/mL TGF-β for 3 h. VEGF and α-Tubulin protein levels were analyzed by western blot (left panel). VEGF mRNA levels were determined by western blot and RT-qPCR (right panel). **(C)** KGN cells were pre-treated with DMSO or 25 µM EGCG for 24 h, then treated with 5 ng/mL TGF-β for 30 min. Western blot analysis was performed to assess phosphorylated and total SMAD2 protein levels, with α-Tubulin serving as a loading control. **(D)** Phosphorylated and total SMAD3 protein levels were also analyzed under the same conditions using western blot. **(E)** KGN cells were transfected with 50 nM control siRNA (si-Ctrl) or SMAD4 siRNA (si-SMAD4) for 48 h, followed by exposure to 25 µM EGCG for 30 min. Protein levels of VEGF, SMAD4, and α-Tubulin were evaluated by western blot. Results are presented as the mean ± SEM of at least three independent experiments. Significant differences are indicated by asterisks (*p < 0.05, **p < 0.01).

TGF-β, a multifunctional cytokine involved in various physiological and pathological processes, including angiogenesis and tissue remodeling, plays a key role in stimulating VEGF expression and secretion in human granulosa-lutein cells via the classical SMAD signaling pathway^36^. This stimulation enhances angiogenesis and vascular permeability, suggesting a potential contribution to OHSS. The classical SMAD pathway is initiated when TGF-β ligands bind to cell surface receptors, such as the TGF-β type 2 receptor (TβRII). This interaction triggers the phosphorylation of receptor-regulated SMADs (R-SMADs), including SMAD2 and SMAD3. The phosphorylated R-SMADs form complexes with SMAD4 and translocate into the nucleus, where they interact with transcriptional partners to regulate gene expression^37^. EGCG has been shown to inhibit TGF-β signaling by directly interacting with TβRII 38. This interaction plays a crucial role in modulating TGF-β signaling pathways and has potential therapeutic implications for managing OHSS. Molecular docking analysis, illustrated in Figure 3A, demonstrates the potential binding position of EGCG with TβRII. Previous research confirmed that treating KGN cells with TGF-β for 3 hours significantly increased VEGF expression^36^. However, pre-treatment with varying concentrations of EGCG prior to TGF-β exposure significantly reduced VEGF expression at both the mRNA and protein levels (Figure 3B). This suppression highlights the effectiveness of EGCG in counteracting the TGF-β-induced increase in VEGF. The activation of TGF-β involves phosphorylation of SMAD2 and SMAD3, which subsequently bind to the common mediator SMAD4. Western blot analysis confirmed the phosphorylation and activation of SMAD2 (Figure 3C) and SMAD3 (Figure 3D) following TGF-β treatment. Pre-treatment with EGCG significantly reduced the levels of phosphorylated SMAD2 and SMAD3, demonstrating its inhibitory effect on their activation. To further explore the role of the classical SMAD signaling pathway in the reduction of TGF-β-induced VEGF expression by EGCG, endogenous SMAD4 expression was silenced using specific siRNA (si-SMAD4). Silencing SMAD4 attenuated the ability of EGCG to suppress VEGF expression, confirming the involvement of SMAD4 in this regulatory mechanism.

### EGCG and Pro-EGCG exhibit similar cytotoxic effects and effectively reduce the expression of VEGF and VEGFR-2

**Figure 4:**
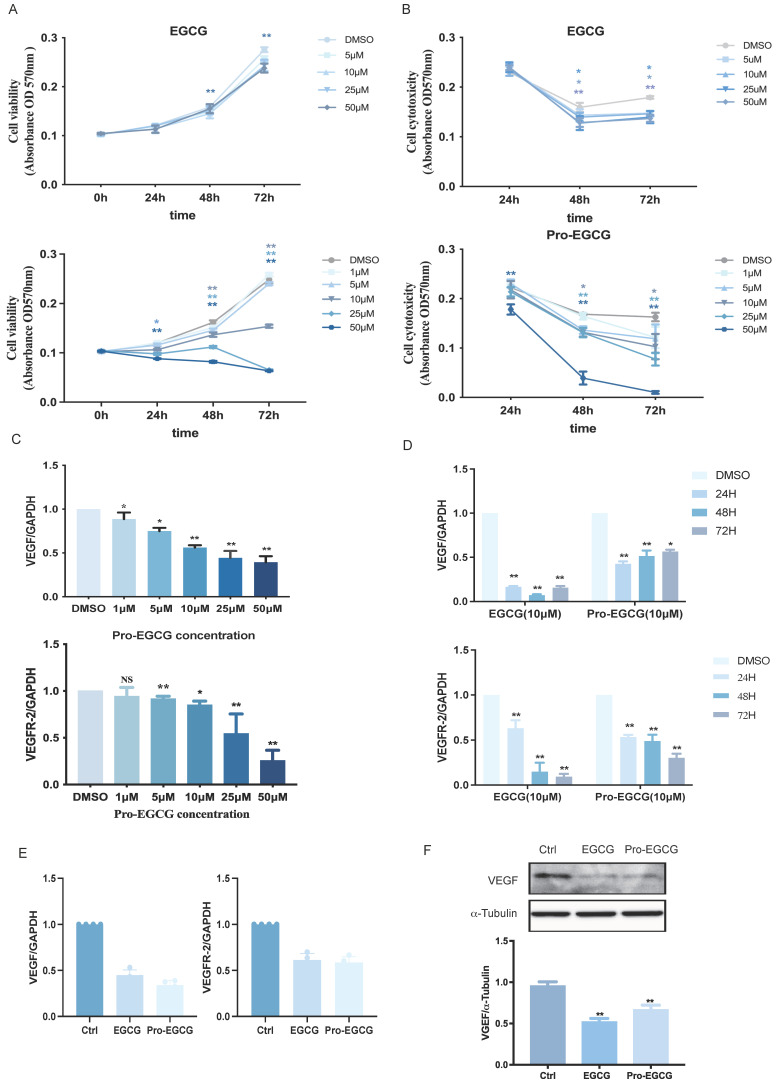
** EGCG and Pro-EGCG exhibit similar cytotoxic effects and effectively reduce VEGF and VEGFR-2 expression. (A)** KGN cells were treated with vehicle control (DMSO) or varying concentrations of EGCG (upper panel) or Pro-EGCG (lower panel) for 0, 24, 48, and 72 h. Cell viability was assessed using the MTT assay. **(B)** Cytotoxicity was further evaluated under the same conditions as in (A) using the MTT assay. **(C)** KGN cells were treated with DMSO or different concentrations of Pro-EGCG for 24 h, and VEGF and VEGFR-2 mRNA levels were quantified by RT-qPCR. **(D)** KGN cells were exposed to DMSO, 10 µM EGCG, or 10 µM Pro-EGCG for 24, 48, and 72 h. VEGF and VEGFR-2 mRNA levels were analyzed by RT-qPCR. **(E)** Primary hGL cells were treated with DMSO, 10 µM EGCG, or 10 µM Pro-EGCG for 24 h, and VEGF and VEGFR-2 mRNA levels were determined by RT-qPCR. **(F)** Protein levels of VEGF in primary hGL cells were examined by western blot after treatment with DMSO, 10 µM EGCG, or 10 µM Pro-EGCG. Results are presented as the mean ± SEM from at least three independent experiments. Significant differences are denoted by asterisks (*p < 0.05, **p < 0.01).

EGCG is known to exhibit poor bioavailability due to low absorption, rapid metabolism, and reduced stability. To address these limitations, a modified variant, Pro-EGCG, has been developed, offering improved stability, bioavailability, and biological activity *in vivo*. Pro-EGCG has demonstrated significant inhibitory effects on the development, growth, and angiogenesis of experimental endometriosis in mice. In this study, the effects of EGCG and Pro-EGCG on KGN cell proliferation were assessed using the MTT assay at various concentrations and time points (Figure 4A). EGCG effectively suppressed KGN cell proliferation, particularly at higher concentrations and longer durations. Notably, Pro-EGCG exhibited an even stronger proliferation-suppressive effect compared to EGCG. The cytotoxicity of EGCG and Pro-EGCG on KGN cells was also evaluated using the MTT assay (Figure 4B). EGCG showed cytotoxic effects starting at 25 µM after 48 hours. In contrast, Pro-EGCG demonstrated significant cytotoxicity at lower concentrations and shorter durations, highlighting its greater potency.

The regulatory effects of Pro-EGCG on VEGF and VEGFR-2 expression in KGN cells were examined in a concentration-dependent manner over 24 hours (Figure 4C). Compared to the inhibitory effects of EGCG on VEGF and VEGFR-2 (Figure 1F), Pro-EGCG exhibited greater efficiency, suppressing VEGF and VEGFR-2 RNA expression at lower concentrations. The time-dependent effects of EGCG and Pro-EGCG on VEGF expression were also evaluated at the same concentration (Figure 4D). Both compounds inhibited VEGF expression at all time points; however, EGCG reached its maximum effect at 48 hours, while Pro-EGCG achieved similar results within 24 hours. Both compounds suppressed VEGFR-2 expression across all time points in a time-dependent manner. Further analysis was conducted on primary human granulosa cells treated with DMSO, EGCG, or Pro-EGCG. These treatments significantly reduced VEGF and VEGFR-2 expression at both the RNA (Figure 4E) and protein levels (Figure 4F).

### EGCG or Pro-EGCG alleviate OHSS in rats

**Figure 5:**
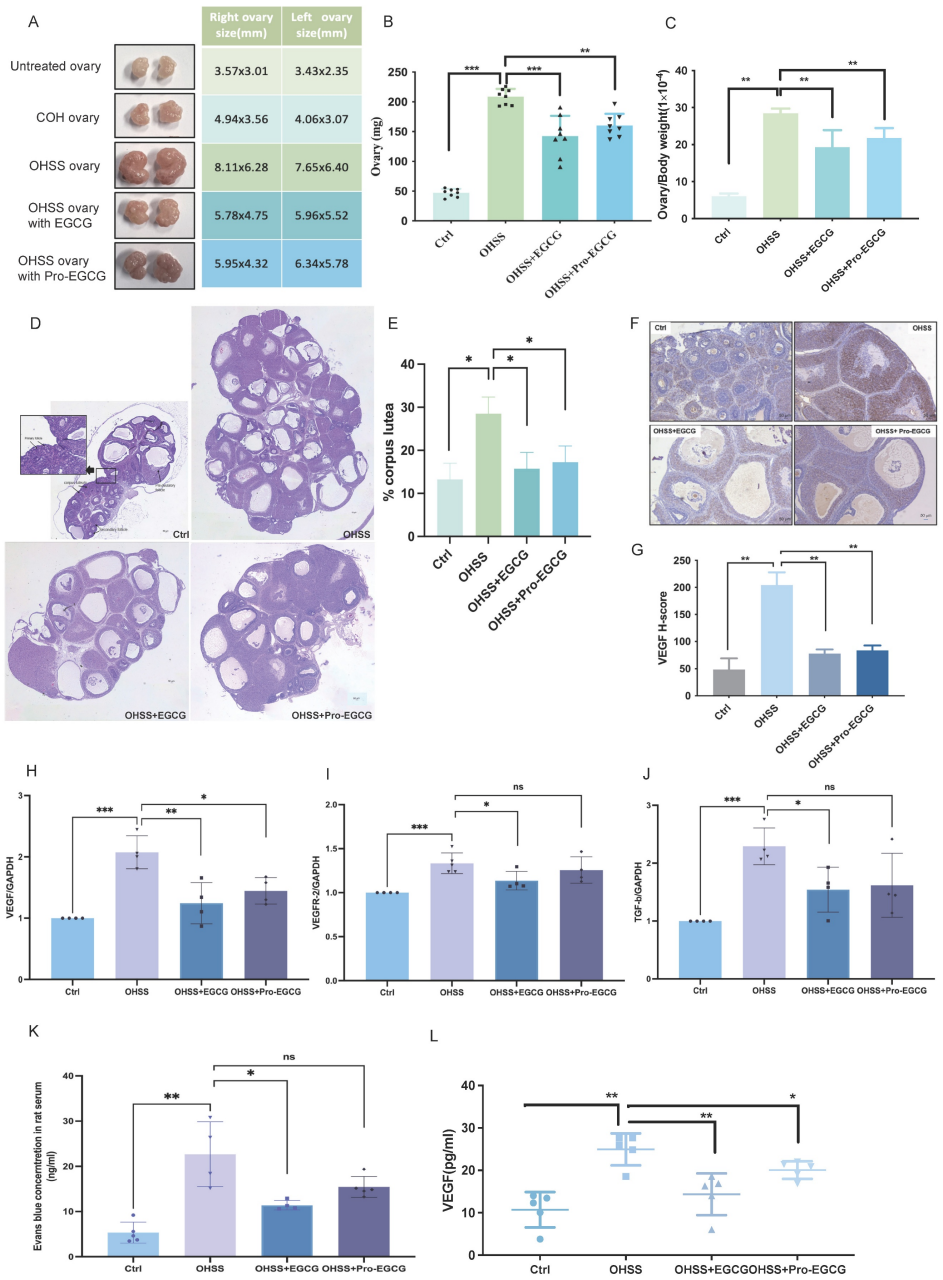
** EGCG and Pro-EGCG alleviate OHSS development in rats. (A)** Representative images of ovaries from each experimental group. **(B, C)** Ovarian weight (B) and ovarian weight normalized to body weight (C) were measured after euthanasia. **(D)** Representative H&E-stained ovarian sections. Images were captured at 400× magnification; scale bars represent 50 μm. **(E)** Percentage of corpora lutea in ovarian sections was quantified (n = 4 per group). **(F)** Representative IHC staining of ovarian tissues for VEGF, with images captured at 400× magnification; scale bars represent 50 μm. **(G)** Quantitative analysis of VEGF protein expression in ovarian tissues based on IHC staining. VEGF expression was evaluated as positive staining intensity. **(H-J)** mRNA levels of VEGF (H), VEGFR-2 (I), and TGF-β (J) in rat ovaries were quantified by RT-qPCR. **(K)** Levels of Evans Blue dye in peritoneal fluid were measured at OD620 nm across different groups. **(L)** Serum VEGF protein levels were quantified using ELISA. Results are presented as the mean ± SEM from at least three independent experiments. Significant differences are indicated by asterisks (*p < 0.05, **p < 0.01).

To further explore the modulatory effects of EGCG and Pro-EGCG on OHSS pathogenesis, we developed an OHSS rat model and evaluated these compounds as potential treatments. Consistent with previous findings, induction of OHSS led to significant ovarian enlargement and increased ovarian weight^39^,^40^. Treatment with EGCG or Pro-EGCG effectively alleviated the severity of these symptoms (Figures 5A-C). Histological analysis confirmed a higher number of corpora lutea in OHSS rats compared to the control group, consistent with earlier studies^40^. Treatment with EGCG or Pro-EGCG significantly reduced the number of corpora lutea in OHSS rats (Figures 5D, 5E).

Immunohistochemistry revealed a marked upregulation of VEGF expression in granulosa cells, luteal cells, and stromal cells within the ovaries of OHSS rats. Administration of EGCG or Pro-EGCG significantly attenuated VEGF expression in these ovarian tissues (Figures 5F, 5G). RT-qPCR analysis further demonstrated that VEGF mRNA levels were elevated in the ovaries of OHSS rats. This upregulation was significantly suppressed by treatment with EGCG or Pro-EGCG (Figure 5H). Similarly, mRNA levels of VEGFR-2 and TGF-β were significantly increased in OHSS rat ovaries but were effectively reduced following treatment with EGCG or Pro-EGCG (Figures 5I, 5J). To validate the effects of EGCG and Pro-EGCG on vascular permeability in the OHSS model, Evans Blue dye was used. Vascular permeability was significantly elevated in OHSS rats compared to controls. Treatment with EGCG or Pro-EGCG significantly reduced this increase in vascular permeability (Figure 5K). Lastly, VEGF levels in rat serum were quantified using an ELISA assay. Serum VEGF levels were significantly higher in OHSS rats, consistent with the pathogenesis of the condition (Figure 5L). Importantly, administration of EGCG or Pro-EGCG led to a marked reduction in serum VEGF levels.

### RNA sequencing results in KGN cells Treated with EGCG or Pro-EGCG

**Figure 6:**
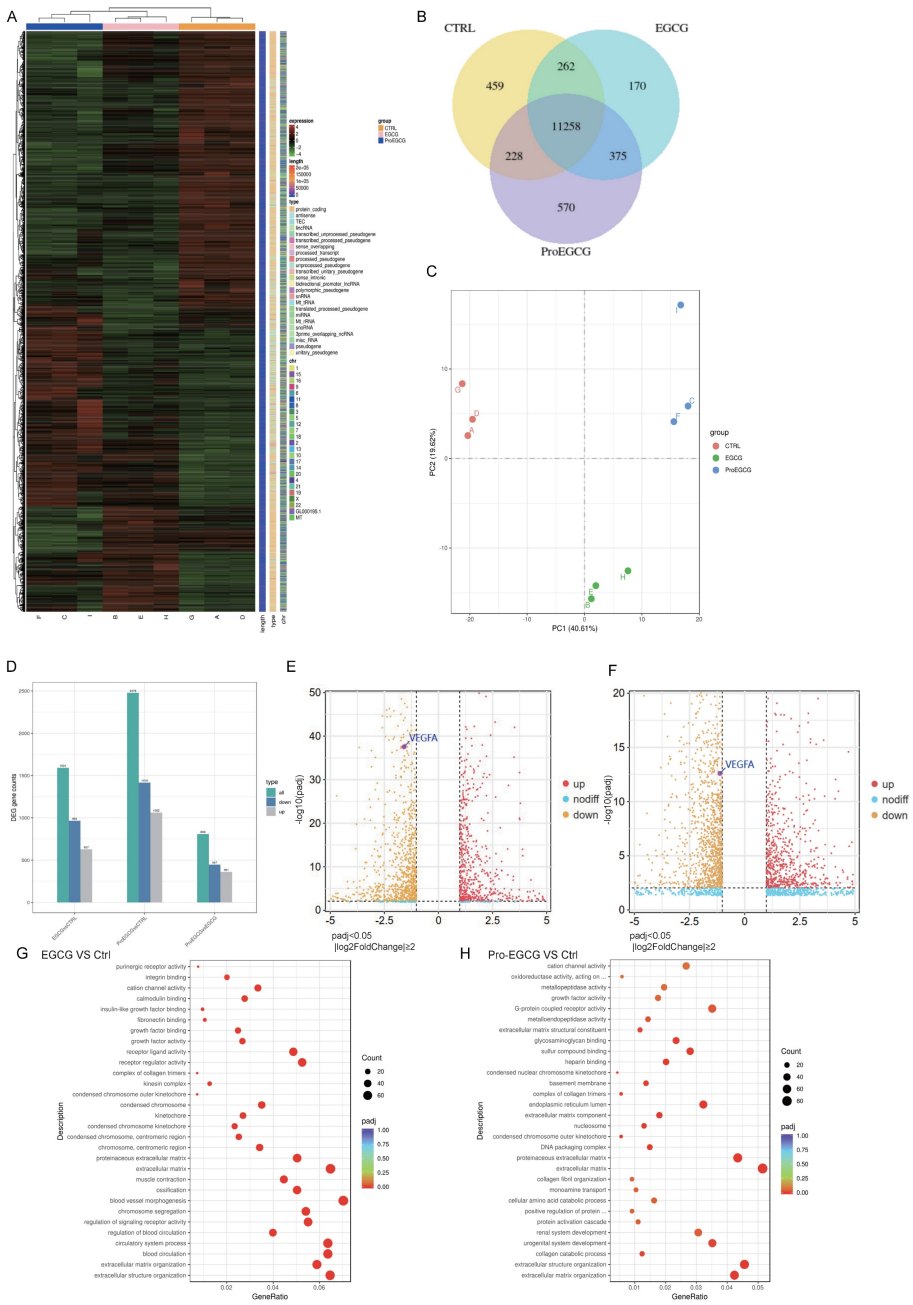
** RNA-sequencing results in KGN cells treated with EGCG and Pro-EGCG. (A)** Heatmap displaying the top 200 most upregulated and downregulated genes. **(B)** Venn diagram illustrating co-expression of genes across different treatment groups. **(C)** Principal component analysis (PCA) of all samples to visualize variance and clustering. **(D)** Bar chart showing the number of differentially expressed genes (DEGs) in each group comparison.** (E)** Volcano plot depicting gene expression differences between EGCG-treated and control cells.** (F)** Volcano plot showing gene expression differences between Pro-EGCG-treated and control cells. (G) GO (Gene Ontology) enrichment analysis scatter plot for EGCG-treated versus control cells, highlighting GO terms with significant enrichment (padj < 0.05). **(H)** GO enrichment analysis scatter plot for Pro-EGCG-treated versus control cells, showing significantly enriched GO terms (padj < 0.05).

To validate the transcriptomic changes in KGN cells following treatment with EGCG and Pro-EGCG, we subjected these KGN cells treated by EGCG or Pro-EGCG to RNA-Seq analysis. The top 200 upregulated and downregulated genes were represented in Figure 6A. The Venn diagram illustrates that 11,258 genes were co-expressed across different groups (Figure 6B). The Principal Component Analysis (PCA) results of the samples highlight the robustness and reliability of the observed differences in gene expression among the control and treated groups (Figure. 6C). The statistics of the number of differential genes (including up-regulation and down-regulation) for each compare group and the threshold for screening are shown in Figure 6D. Comparing the EGCG-treated group to the control group, the results revealed that 627 genes were up-regulated, while 964 genes were down-regulated. In the case of the Pro-EGCG-treated group compared to the control group, 1062 genes were up-regulated, while 1416 genes were down-regulated. These findings provide insights into the specific gene expression alterations induced by EGCG and Pro-EGCG treatments in relation to the control group. The volcano plot depicting the comparison of gene expression levels between EGCG-treated and control cells was highlighted the expression of VEGFa (Figure 6E), same in volcano plot depicting the comparison of gene expression levels between Pro-EGCG treated and control cells (Figure 6F). GO, an acronym for Gene Ontology, represents a prominent bioinformatics framework aimed at standardizing the delineation of gene attributes across diverse species. Comprising three primary categories, namely cellular component, molecular function, and biological process, GO facilitates a comprehensive understanding of gene functionalities. Following a rigorous GO enrichment analysis, the top 30 GO Terms, deemed most statistically significant, were meticulously curated for presentation and analysis (Figure 6E, 6G). The RNA-seq analysis provides further confirmation that both EGCG and Pro-EGCG treatments significantly reduce the expression of VEGF, corroborating our earlier study.

## Discussion

While the occurrence of severe OHSS is relatively infrequent, it persists as a substantial and concerning complication associated with *in vitro* fertilization procedures. The incident of OHSS is heavily monitored by all local ART regulatory bodies. The pathophysiology of OHSS, although extensively studied, remains enigmatic, resulting in predominantly empirical and anticipatory clinical management approaches. A range of strategies has been deployed to preclude the onset of OHSS, including reducing gonadotropin dosages, employing GnRH antagonists for ovulation triggering, implementing cryopreservation techniques, considering cycle cancellation, and others^41^-^43^. Nonetheless, none of these approaches have proven entirely effective in providing comprehensive protection against the development of OHSS. Consequently, the pursuit of an optimal pharmaceutical intervention to proactively forestall this potentially life-threatening complication remains a paramount challenge within the domain of assisted reproductive technologies. Recognizing the central involvement of VEGF in OHSS pathogenesis, interventions aimed at VEGF modulation have been harnessed as a preventive measure against this syndrome^19^. To the best of our knowledge, this is the first study to demonstrate the therapeutic potential of EGCG and its derivative, Pro-EGCG, in alleviating OHSS, providing novel evidence of their efficacy in both *in vitro* and *in vivo* models. Our findings reveal that EGCG treatment suppresses VEGF production by inhibiting the TGF-β/Smad and PKA-CREB signaling pathways, highlighting a previously unrecognized mechanism. These results establish EGCG as a promising adjuvant therapy for OHSS, offering a strong mechanistic foundation for its potential clinical application.

This study delved into the therapeutic potential of EGCG and its modified form, Pro-EGCG, as agents for OHSS treatment. EGCG is a naturally occurring compound found in green tea and has a well-established safety profile, making it a promising candidate for therapeutic use. Its accessibility and cost-effectiveness contribute to its appeal as a potential treatment. Investigations revealed that EGCG exerts inhibitory effects on key factors such as VEGF and its receptor VEGFR-2, which are implicated in the pathogenesis of OHSS. This suggests that EGCG may help mitigate the angiogenic and vascular permeability processes responsible for OHSS development. In essence, the multifaceted benefits of EGCG, including safety, affordability, and capacity to target key factors in OHSS pathophysiology, underscore its potential as a valuable therapeutic approach for addressing this complex syndrome.

A pivotal aspect of the study involved deciphering the mechanisms through which EGCG exerts an inhibitory effect on VEGF expression. Findings indicated that EGCG interacts with the 67LR and subsequently triggers the protein kinase A (PKA)/cAMP-responsive element-binding protein (CREB) signaling pathway, resulting in the suppression of VEGF expression. While previous research suggested that TGF-β stimulation can enhance VEGF expression through the classical SMAD signaling pathway, this study, for the first time, shed light on the specific molecular events through which EGCG inhibits VEGF in KGN cells and primary human granulosa cells. The interaction between TGF-β and EGCG was investigated to understand how TGF-β might be involved in EGCG effects. TGF-β is a multifunctional cytokine known to participate in various physiological and pathological processes, including angiogenesis and tissue remodeling. Previous studies confirmed that EGCG binds to TGF-β type II receptor (TβRII), effectively hindering TGF-β actions by disrupting its interaction with TβRII in MRC-5 cells^38^. Many studies also investigated EGCG inhibitory effects on TGF-β through SMAD or ERK1/2 signaling pathways 44,45.

This study establishes a novel link between EGCG capability to inhibit VEGF expression and the classical SMAD signaling pathway associated with TGF-β. The results suggest that EGCG capacity to interact with TGF-β receptors, particularly TβRII, enables it to interfere with the activation of SMAD2 and SMAD3. This interference disrupts their translocation into the nucleus, where they typically engage with transcriptional partners to regulate gene expression. Consequently, this interference results in downstream effects on the expression of genes pertinent to angiogenesis, such as VEGF and its receptor VEGFR-2, both of which are pivotal in the context of OHSS pathogenesis. This elucidation of the precise mechanisms underpinning VEGF modulation provides a foundational understanding of the potential therapeutic effects of EGCG in OHSS.

Pro-EGCG demonstrated robust proteasome inhibition and induction of cell death in cancer cells^46^. Its potency surpassed that of EGCG in inhibiting proliferation, transforming activity, and inducing apoptosis in various human cancer cell types, including breast, prostate, leukemic, and simian virus 40-transformed cells^47^. Consistent with previous research, this study revealed that Pro-EGCG exhibited superior efficacy compared to EGCG in inhibiting proliferation and inducing cytotoxicity in KGN cells. These findings further support the notion that Pro-EGCG possesses potent cell death-inducing properties and outperforms EGCG in terms of efficiency. Additionally, this study confirmed that Pro-EGCG effectively inhibited the expression of VEGF and its receptor in KGN cells. Notably, the inhibitory effects on VEGF and its receptor were observed at lower concentrations compared to EGCG, providing further evidence of its enhanced efficiency. Previous studies demonstrated that Pro-EGCG and EGCG do not affect ovarian follicles in mice ovaries, suggesting that this natural anti-angiogenic agent may not disrupt normal ovulation^27^. Consequently, Pro-EGCG emerges as a stable and potent green tea polyphenol with the potential to serve as a novel anti-angiogenic agent for OHSS.

The time-dependent inhibitory effects of EGCG and Pro-EGCG on VEGF and VEGFR-2 expression in KGN cells are noteworthy. The differential optimal time points for each compound indicate that their mechanisms of action may involve distinct pathways and kinetics. Therefore, further investigations are warranted to elucidate the specific mechanisms of action for Pro-EGCG in the context of OHSS. Additionally, due to the limitations imposed by the sample size in this study, additional experiments are needed to confirm the effects of Pro-EGCG in primary hGL cells. Interestingly, the study revealed that Pro-EGCG, despite its enhanced stability and bioavailability, did not exhibit a significantly stronger therapeutic effect in the OHSS animal model when compared to EGCG. This unexpected finding raises important questions about the nuanced interactions of these compounds with the complex biological pathways implicated in OHSS. Potential explanations for this observation could include differences in pharmacokinetics, tissue distribution, or specific interactions with the OHSS pathogenic cascade. Further research in these areas will provide a more comprehensive understanding of the therapeutic potential and underlying mechanisms of Pro-EGCG in the management of OHSS.

Despite their potential, EGCG and Pro-EGCG have several limitations. The poor bioavailability and rapid metabolism of EGCG may restrict its clinical utility^27^,^48^, while the long-term safety and pharmacokinetics of Pro-EGCG remain insufficiently explored. Both compounds exhibit dose-dependent cytotoxicity and broad-spectrum activity on signaling pathways, including PI3K/AKT and MAPK, which raises concerns about potential off-target effects 49. Additionally, translating findings from animal models to human applications presents challenges due to interspecies differences in drug metabolism and ovarian physiology. Future research should focus on conducting clinical trials to assess the safety, efficacy, and pharmacodynamics of Pro-EGCG in human populations.

In conclusion, our study highlights the therapeutic potential of EGCG for mitigating OHSS and elucidates its mechanisms of action, particularly its modulation of VEGF expression. While Pro-EGCG offers improved stability and potency, its similar efficacy to EGCG in OHSS warrants further investigation. These findings contribute to the growing understanding of OHSS pathogenesis and offer a foundation for refining therapeutic strategies in assisted reproductive technologies.

## Supplementary Material

Supplementary tables.
