# Supplementary material for: The Therapeutic Potential of EGCG and Pro-EGCG in Mitigating Ovarian Hyperstimulation Syndrome: Unraveling the Modulatory Mechanism through the VEGF Pathway
Source: Int J Biol Sci. 2025 Apr 22;21(7):3045–60. doi: 10.7150/ijbs.98653 (PMC12080398; doi:10.7150/ijbs.98653)
Supplement: Supplementary file 1 — Supplementary tables. [file ijbsv21p3045s1.pdf]

Supplemental Table 1: The Antibodies used for Western blot.

Name	Primary/Secondary	Type	Host	Source	Catalogue Number	Dilution
**Anti-α-tubulin**	Primary	Monoclonal	Mouse	Santa Cruz Biotechnology	#sc-23948	1:5000
**Anti-caspase 3**	Primary	Monoclonal	Rabbit	Abcam	#ab32351	1:2000
**Anti-VEGF**	Primary	Monoclonal	Mouse	ThermoFisher	# MA5-13182	1:1000
**Anti-phospho-CREB**	Primary	Monoclonal	Rabbit	Cell Signaling Technology	#9198	1:1000
**Anti-CREB**	Primary	Monoclonal	Rabbit	Cell Signaling Technology	#9197	1:1000
**Anti-67-kDa laminin receptor**	Primary	Monoclonal	Rabbit	Abcam	#ab133645	1:1000
**Anti-Smad2**	Primary	Monoclonal	Rabbit	Cell Signaling Technology	#5339	1:500
**Anti-phospho-Smad2**	Primary	Monoclonal	Rabbit	Cell Signaling Technology	#18338	1:500
**Anti-Smad3**	Primary	Monoclonal	Rabbit	Cell Signaling Technology	#9523	1:500
**Anti-phospho-Smad3**	Primary	Monoclonal	Rabbit	Cell Signaling Technology	#9520	1:500
**Anti-rabbit**	Secondary	N/A	Goat	Abcam	#ab6721	1:2000
**Anti-mouse**	Secondary	N/A	Goat	Abcam	#ab6789	1:2000

Supplemental Table 2: The primer sequences used for real-time PCR.

Gene	Forward/Reverse	Sequence
hGAPDH	Forward	5′- ATGGAAATCCCATCACCATCTT-3′
	Reverse	5′-CGCCCCACTTGATTTTGG-3′
hVEGF	Forward	5′- CCCACTGAGGAGTCCAACAT-3′
	Reverse	5′- TGCATTCACATTTGTTGTGC-3′
hTGFβ1	Forward	5′- TACCTGAACCCGTGTTGCTCTC-3′
	Reverse	5′- GTTGCTGAGGTATCGCCAGGAA-3′
hVEGFR2	Forward	5′- CATCCAGTGGGCTGATGACC-3′
	Reverse	5′- TTCGCAGGGATTCTGACACG-3′
rGAPDH	Forward	5′- GACATGCCGCCTGGAGAAAC-3′
	Reverse	5′- AGCCCAGGATGCCCTTTAGT-3′
rVEGF	Forward	5′- ACGAAAGCGCAAGAAATCCC-3′
	Reverse	5′- TAACTCAAGCTGCCTCGCC-3′
rVEGFR2	Forward	5′- AAGCAAATGCTCAGCAGGAT-3′
	Reverse	5′- GAGGTAGGCAGGGAGAGTCC-3′
rTGFβ1	Forward	5′- TCAACTGTGGAGCAACACGT-3′
	Reverse	5′- CACTCAGGCGTATCAGTGGG-3′
